# Effect of prognostic classification on temsirolimus efficacy and safety in patients with relapsed or refractory mantle cell lymphoma: a retrospective analysis

**DOI:** 10.1186/s40164-015-0006-1

**Published:** 2015-04-11

**Authors:** Georg Hess, Bertrand Coiffier, Michael Crump, Christian Gisselbrecht, Fritz Offner, Jorge Romaguera, Lisa Kang, Pádraig J Moran

**Affiliations:** Department of Hematology/Oncology, Johannes Gutenberg University, Langenbeckstr. 1, Mainz, DE 55101 Germany; Centre Hospitalier Lyon Sud, Hospices Civils de Lyon, Pierre-Benite, France; Princess Margaret Hospital, Toronto, ON Canada; Hôpital Saint-Louis, Paris, France; Ghent University Hospital, Ghent, Belgium; The University of Texas MD Anderson Cancer Center, Houston, TX USA; Pfizer Inc, Cambridge, MA USA; Prizer Inc, Dublin, Ireland

**Keywords:** Mantle cell lymphoma, Prognostic, Risk, Temsirolimus, Efficacy, Safety

## Abstract

**Background:**

Temsirolimus, a selective inhibitor of the mammalian target of rapamycin, has demonstrated clinical benefit versus investigator’s choice (INV) of therapy in patients with relapsed/refractory mantle cell lymphoma (MCL).

**Methods:**

This post hoc study retrospectively assigned simplified Mantle Cell Lymphoma International Prognostic Index (MIPI) scores (ie, secondary MIPI) based on parameters at the time of randomization in patients with MCL (N = 162) who received temsirolimus 175 mg once weekly for 3 weeks followed by once-weekly 75 mg or 25 mg or the INV of active therapy. Outcomes were analyzed according to the low-, intermediate- or high-risk category.

**Results:**

Patient distribution by MIPI risk category was 31%, 39%, and 30% in the low-, intermediate-, and high-risk groups, respectively. Among patients in all categories, objective response rate (complete response + partial response) was higher in patients in the temsirolimus 175/75-mg group versus the INV group, respectively: 42% versus 0% (low-risk); 33% versus 5% (intermediate-risk); 10% versus 0% (high-risk). Median progression-free survival was significantly longer with temsirolimus 175/75 mg versus INV, respectively, in patients with intermediate (4.3 vs 1.9 months; *P* = 0.035) or high (4.5 vs 1.6 months; *P* = 0.0025) risk, and a trend toward improvement was observed in patients with low risk (5.3 vs 2.6 months; *P* = 0.091). Improvement in median overall survival was observed with temsirolimus 175/75 mg versus INV in low-risk patients (18.0 vs 10.5 months, respectively; *P* = 0.069).

**Conclusions:**

This analysis suggests that, compared with INV, temsirolimus demonstrated benefit in all MIPI risk categories in patients with MCL. In all treatment groups, patients with high secondary MIPI scores at baseline faced a dismal prognosis.

**Trial registration:**

ClinicalTrials.gov NCT00117598.

**Electronic supplementary material:**

The online version of this article (doi:10.1186/s40164-015-0006-1) contains supplementary material, which is available to authorized users.

## Introduction

The introduction of new therapeutic strategies has led to improvements in response rates and/or longer progression-free survival (PFS) in individuals who are newly diagnosed with mantle cell lymphoma (MCL) [1-4]. However, outcomes remain poor (median survival, <2 years) in patients with MCL that has relapsed or is refractory to first-line regimens.

Temsirolimus is a selective inhibitor of the mammalian target of rapamycin (mTOR) signaling protein, which can be aberrantly activated in MCL [5-7]. Several clinical studies, including one randomized controlled trial, have demonstrated that temsirolimus has single-agent activity in patients with relapsed or refractory MCL [8-10]. In Europe, temsirolimus is registered for treatment of adults with relapsed or refractory MCL [11]. Consensus guidelines from the European Society for Molecular Oncology support the use of temsirolimus in advanced relapses (greater than second line) and especially in relapsed nonfit patients [12].

In the pivotal multicenter, randomized phase III trial [9], 162 patients with relapsed or refractory MCL were randomized to receive temsirolimus 175 mg once weekly for 3 weeks followed by once-weekly doses of either 75 mg (175/75-mg regimen) or 25 mg (175/25-mg regimen), or the investigator’s choice (INV) of active therapy. Treatment in the INV arm consisted primarily of single-agent gemcitabine (42%) or fludarabine (27%), along with a number other single agents. Temsirolimus 175/75 mg significantly prolonged median PFS compared with INV (4.8 vs 1.9 months; hazard ratio [HR] = 0.44; *P* = 0.0009) [9]. The lower dose of temsirolimus (175/25 mg) also showed longer median PFS (3.4 months) versus INV, but this difference was not significant. Exploratory subgroup analyses revealed no significant differences in PFS benefit based on sex, Karnofsky performance score (KPS) at baseline, stage of disease at diagnosis, bone marrow involvement, number of extranodal sites or number of prior anticancer regimens [9]. Overall survival was longer with temsirolimus 175/75 mg than with INV, but this difference was not significant (12.8 vs 9.7 months; *P* = 0.3519) [9].

Patients in the phase III trial were not assigned a risk category at baseline, owing to the absence of a robust, validated prognostic scoring system for MCL. Since then, the simplified Mantle Cell Lymphoma International Prognostic Index (MIPI) was validated and was shown to have high prognostic significance for newly diagnosed patients, but it is not formally used for patients with relapsed or refractory disease [13-16]. The objectives of the present study were to understand whether certain MIPI risk groups benefit more from treatment with temsirolimus, or if there are patients unlikely to respond to treatment, and to evaluate the potential utility of MIPI in the context of relapsed/refractory MCL. In this study, simplified MIPI criteria were used to retrospectively assign prognostic scores to patients with relapsed/refractory MCL (ie, secondary MIPI) at the time of their trial enrollment, and outcomes were analyzed according to risk category.

## Results and discussion

### Patients

Baseline characteristics for the patients in the randomized phase III trial are provided in a prior publication [9]. In brief, median age was 67 years (range 39–88), 81% of patients were male, 85% had KPS ≥80, and 46% had bone marrow involvement. The three treatment groups were generally well balanced with respect to baseline characteristics. An exception was that blastoid histology was noted for zero, nine, and four patients in the temsirolimus 175/75-mg, 175/25-mg, and INV groups, respectively. The median number of prior regimens was three in both temsirolimus groups and four in the INV group. Approximately one third of the patients had undergone autologous hematopoietic stem-cell transplantation following high-dose therapy.

All 162 patients were assigned simplified MIPI scores based on baseline characteristics at their time of enrollment. Of the 162 patients, 14 were missing one MIPI value, one patient was missing two MIPI values, and two patients were missing three MIPI values. Patient distribution was relatively even across the low (n = 51), intermediate (n = 63), and high (n = 48) MIPI risk categories (Table 1).

**Table 1:** **Distribution of patients within each treatment arm by simplified MIPI risk category** [13,14]

**Patients**	**Low risk**	**Intermediate risk**	**High risk**
	**n (%)**	**n (%)**	**n (%)**
All patients (N = 162)	51 (31)	63 (39)	48 (30)
Temsirolimus 175/75 mg (n = 54)	14 (26)	24 (44)	16 (30)
Temsirolimus 175/25 mg (n = 54)	15 (28)	18 (33)	21 (39)
INV therapy (n = 54)	22 (41)	21 (39)	11 (20)

MIPI = Mantle Cell Lymphoma International Prognostic Index; INV = Investigator’s choice.

MIPI distributions were comparable, with the exception of a greater proportion of intermediate-risk patients in the temsirolimus 175/75-mg arm than in the temsirolimus 175/25-mg arm (44% and 33%, respectively; Table 1). The INV arm had a greater proportion of low-risk patients and a lower proportion of high-risk patients relative to both of temsirolimus treatment arms (Table 1). Of the 13 patients with blastoid variant, nine were classified as high risk (175/25 mg [n = 7] and INV [n = 2]) and four were classified as intermediate risk (175/25 mg [n = 2] and INV [n = 2]).

### Treatment response

Assessment of best response was available for 123 (76%) patients. Because the distribution of the 39 patients who did not have tumor assessments available was imbalanced across these small subsets, tumor responses are reported for the evaluable population. Among patients in all MIPI categories, objective response rate (complete response [CR] + partial response [PR]) was higher in the temsirolimus 175/75-mg group compared with the INV arm, respectively: 42% versus 0% (low risk), 33% versus 5% (intermediate risk), and 10% versus 0% (high risk). Complete response was achieved in one patient in each of the two treatment groups; both were classified as intermediate MIPI. In the temsirolimus 175/25-mg group, no CR was observed and the objective response rate by MIPI category was 15% (low risk), 7% (intermediate risk), and 0% (high risk).

Stable disease (SD) is not a standard end point in MCL studies; however, patients can be asymptomatic for long periods. Furthermore, because commonly used regimens administered in the INV group achieved almost no objective responses (one CR, no PR) in these heavily pretreated patients, disease stabilization may be even more relevant in this setting. When analyzed by MIPI categories, higher clinical benefit rates (CBRs), calculated here as CR + PR + SD ≥8 weeks, were observed in the temsirolimus 175/75-mg arm versus the INV arm for patients in all risk groups (Table 2). Among low-risk patients, CBR was 75%, 77%, and 36% with temsirolimus 175/75 mg, temsirolimus 175/25 mg, and INV, respectively. Intermediate-risk patients had CBRs of 61%, 33%, and 40% in the respective treatment groups. For high-risk patients, CBRs were 80% and 33% in the temsirolimus 175/75 and 175/25 arms, respectively, whereas no patient achieved clinical benefit in the INV arm. These results suggest that patients in all secondary MIPI groups may derive some clinical benefit from temsirolimus. In addition, treatment duration was more sustained in patients administered temsirolimus 175/75 mg or 175/25 mg compared with INV-treated patients in all MIPI risk categories (Table 3). Although there could be different reasons why, in patients with SD, the doctor and/or patient decide to continue temsirolimus therapy, the longer treatment duration observed with temsirolimus may provide an indirect sign of clinical benefit.

**Table 2:** Number of evaluable patients (%) with stable disease or better response* by MIPI risk category [13,14]

	**n/N (%) [n]**		
**Treatment**	**Low risk**	**Intermediate risk**	**High risk**
Temsirolimus 175/75 mg	9/12 (75)	11/18 (61)	8/10 (80)
	[5 PR, 4 SD]	[1 CR, 5 PR, 5 SD]	[1 PR, 7 SD]
Temsirolimus 175/25 mg	10/13 (77)	5/15 (33)	5/15 (33)
	[2 PR, 8 SD]	[1 PR, 4 SD]	[5 SD]
INV therapy	4/11 (36)	8/20 (40)	0/9 (0)
	[4 SD]	[1 CR, 7 SD]	

*Disease assessment was based on radiographic review by independent radiologists and review of clinical data by independent oncologists; evaluable patients were those with tumor assessment available.

MIPI = Mantle Cell Lymphoma International Prognostic Index; PR = partial response; SD = stable disease ≥8 weeks; CR = complete response; INV = Investigator’s choice.

**Table 3:** **Treatment duration in each treatment arm by simplified MIPI risk category** [13,14]

**Treatment**	**Low risk**	**Intermediate risk**	**High risk**
**Temsirolimus 175/75 mg**			
n	14	24	16
Mean (std dev), weeks	32.4 (22.0)	21.1 (24.8)	10.5 (8.8)
**Temsirolimus 175/25 mg**			
n	15	18	21
Mean (std dev), weeks	39.0 (42.3)	17.6 (11.4)	11.7 (12.4)
**INV therapy**			
n	21	21	11
Mean (std dev), weeks	8.1 (7.8)	9.8 (10.3)	3.3 (2.4)

MIPI = Mantle Cell Lymphoma International Prognostic Index; std dev = standard deviation; INV = Investigator’s choice.

### Progression-free survival and overall survival

**Figure 1:**
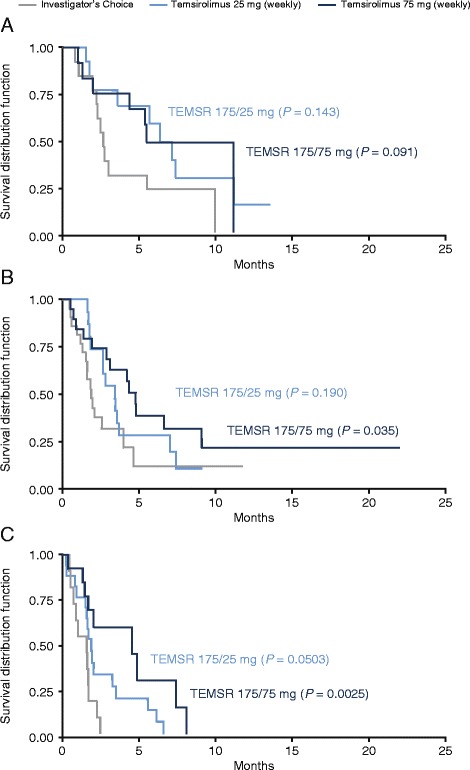
Kaplan-Meier plots of progression-free survival by MIPI risk group. **(A)** Low-risk group, **(B)** intermediate-risk group, and **(C)** high-risk group. Log-rank *P* values given for temsirolimus versus investigator’s choice of therapy. Statistical analyses shown are for illustrative purposes; the phase III trial was not powered to detect differences in progression-free survival for subsets by MIPI risk categories. MIPI = Mantle Cell Lymphoma International Prognostic Index; TEMSR 25 mg = temsirolimus 25 mg administered once weekly after three weekly doses of 175 mg; TEMSR 75 mg = temsirolimus 75 mg administered once weekly after three weekly doses of 175 mg.

Temsirolimus 175/75 mg significantly improved PFS versus INV in intermediate-risk (*P* = 0.035) and high-risk (*P* = 0.0025) patients, and a trend toward improvement was observed in low-risk patients (Figure 1; Table 4). In general, median PFS was longer in patients of all risk groups in the temsirolimus 175/75-mg group than for those in the INV group (Table 4). Among low-risk patients in the temsirolimus 175/25-mg group, median PFS was 3.6 months longer than in the INV group, but this difference was not statistically significant. Similar median PFS were observed for the different patient cohorts (exclusion of patients with blastoid variant, exclusion of patients with missing MIPI values, and exclusion of patients with both blastoid variant and/or at least one missing MIPI value) compared with the analysis of all patients, except for the longer PFS observed in low-risk patients after patients with missing MIPI values were excluded (Table 4). A possible explanation for the longer PFS found after patients with missing MIPI values were excluded from the low-risk group is that this low-risk group might include patients that should have probably been assigned a higher risk level if all MIPI values were recorded for them. By excluding patients with missing MIPI values from this low-risk group, the true value of temsirolimus 175/75 mg becomes more evident.

**Table 4:** **Progression-free survival by MIPI risk category and by analyzed cohort**

**Patient cohort**	**Median PFS (95% CI), months**			**Median OS (95% CI), months**		
	**Temsirolimus 175/75 mg**	**Temsirolimus 175/25 mg**	**INV therapy**	**Temsirolimus 175/75 mg**	**Temsirolimus 175/25 mg**	**INV therapy**
**Low risk**						
All	n = 14	n = 15	n = 22	n = 14	n = 15	n = 22
	5.3 (4.2, 10.9)	6.2 (3.5, 10.9)	2.6 (2.1, 5.4)	18.0 (11.1, 22.3)	14.5 (11.0, NA)	10.5 (6.7, 16.6)
Blastoid excluded	n = 14	n = 15	n = 22	n = 14	n = 15	n = 22
	5.3 (4.2, 10.9)	6.2 (3.5, 10.9)	2.6 (2.1, 5.4)	18.0 (11.1, 22.3)	14.5 (11.0, NA)	10.5 (6.7, 16.6)
Missing values for MIPI excluded	n = 12	n = 13	n = 16	n = 12	n = 13	n = 16
	10.9 (4.2, 10.9)	6.2 (1.9, 10.9)	2.9 (2.4, 9.7)	18.0^*^ (14.1, 22.3)	14.5 (11.0, NA)	9.5 (5.3, 16.6)
Blastoid and missing values excluded	n = 12	n = 13	n = 16	n = 12	n = 13	n = 16
	10.9 (4.2, 10.9)	6.2 (1.9, 10.9)	2.9 (2.4, 9.7)	18.0^*^ (14.1, 22.3)	14.5 (11.0, NA)	9.5 (5.3, 16.6)
**Intermediate risk**						
All	n = 24	n = 18	n = 21	n = 24	n = 18	n = 21
	4.3^*^ (2.9, 9.1)	3.4 (2.6, 3.7)	1.9 (1.6, 4.0)	12.8 (8.1, NE)	8.8 (8.1, 20.6)	12.4 (5.7, 15.1)
Blastoid excluded	n = 24	n = 16	n = 19	n = 24	n = 16	n = 19
	4.3 (2.9, 9.1)	3.4 (1.8, 7.0)	1.9 (1.6, 4.0)	12.8 (8.1, NA)	8.8 (8.1, 20.6)	13.6 (5.7, NA)
Missing values for MIPI excluded	n = 22	n = 18	n = 19	n = 22	n = 18	n = 19
	4.8^*^ (3.1, 9.1)	3.4 (2.6, 3.7)	1.9 (1.3, 4.0)	14.3 (10.0, NA)	8.8 (8.1, 20.6)	12.4 (5.7, 15.1)
Blastoid and missing values excluded	n = 22	n = 16	n = 17	n = 22	n = 16	n = 17
	4.8^*^ (3.1, 9.1)	3.4 (1.8, 7.0)	1.9 (1.3, 4.6)	14.3 (10.0, NA)	8.8 (8.1, 20.6)	12.4 (5.7, NA)
**High risk**						
All	n = 16	n = 21	n = 11	n = 16	n = 21	n = 11
	4.5^†^ (1.7, 7.4)	1.9 (1.5, 3.3)	1.6 (0.7, 1.7)	5.3 (2.0, 9.9)	4.1 (2.0, 7.2)	3.5 (2.0, 4.8)
Blastoid excluded	n = 16	n = 14	n = 9	n = 16	n = 14	n = 9
	4.5^‡^ (1.7, 7.4)	1.9^*^ (1.7, 5.6)	1.6 (0.9, 1.7)	5.3 (2.0, 9.9)	5.1 (3.3, 14.6)	3.7 (3.2, 20.4)
Missing values for MIPI excluded	n = 16	n = 18	n = 11	n = 16	n = 18	n = 11
	4.5^†^ (1.7, 7.4)	1.9^*^ (1.5, 3.3)	1.6 (0.7, 1.7)	5.3 (2.0, 9.9)	4.5 (3.3, 7.2)	3.5 (2.0, 4.8)
Blastoid and missing values excluded	n = 16	n = 12	n = 9	n = 16	n = 12	n = 9
	4.5^‡^ (1.7, 7.4)	1.9^*^ (1.7, 5.6)	1.6 (0.9, 1.7)	5.3 (2.0, 9.9)	5.1 (3.3, 14.6)	3.7 (3.2, 20.4)

^*^ *P* < 0.05 versus INV.

^†^ *P* < 0.005 versus INV.

^‡^ *P* < 0.01 versus INV.

MIPI = Mantle Cell Lymphoma International Prognostic Index; PFS = progression-free survival; CI = confidence interval; OS = overall survival; INV = Investigator’s choice of therapy.

Mean overall survival (OS) in patients treated with temsirolimus 175/75 mg was 18.0 months in low-risk, 12.8 months in intermediate-risk, and 5.3 months in high-risk groups (Table 4). Though not statistically significant, a 7.5 month improvement in median OS was observed in low-risk patients with temsirolimus 175/75 mg compared with INV (18.0 vs 10.5 months; *P* = 0.069). Similar OS results observed for the different patient cohorts with excluded patients (Table 4).

### Safety

For all treatment groups, low-risk patients had longer durations of exposure than did high-risk patients (Table 3). In both temsirolimus arms, the mean duration of treatment by MIPI risk category was: Low > Intermediate > High. For the INV arm, mean treatment durations were Low ≈ Intermediate > High (Table 3).

Among patients treated with temsirolimus 175/75 mg, grade 3 or 4 thrombocytopenia or infection appeared to occur less frequently in low-risk patients than in high-risk patients; no incidence pattern was noted for grade 3 or 4 anemia by risk group. The numbers of patients with at least one dose delay by simplified MIPI risk category are provided online in Additional file 1: Table S1.

### Prognostic factors in relapsed/refractory MCL

For newly diagnosed patients, MIPI classification is increasingly incorporated in clinical trial designs for stratification, as well as in clinical practice to inform treatment decisions [16]. Although not formally established, secondary MIPI in relapse is now frequently used in trials of relapsed/refractory MCL [13,14,17].

In our analysis, secondary MIPI classification, which was calculated for patients with relapsed/refractory MCL, revealed the potential for robust prognostic separation within our randomized phase III trial, although data were not available for all patients [9]. MIPI scores were observed to be good predictors of OS in all three treatment groups in the current study. There was a clear superiority of treatment efficacy in patients with low- and intermediate-risk MIPI categories, whereas patients in the high-risk category did not show improved OS with any of the treatments. Thus, MIPI scores may be applicable for prognostic evaluation and risk-adapted therapeutic strategies in these hard-to-treat patients. Furthermore, including secondary MIPI classification in future trials may make the results from these trials more comparable.

The results of this study should be viewed against its limitations. First, this study was not designed to collect MIPI parameters, which resulted in missing MIPI values for some patients. Second, using the simplified MIPI classification to divide patients into the different risk categories resulted in a lower number of patients available for efficacy evaluation in each arm, thus limiting the statistical power of many of these comparisons.

## Conclusions

In conclusion, temsirolimus induced responses in all risk groups of patients with relapsed/refractory MCL who were retrospectively assigned MIPI scores. In all MIPI risk groups, objective response rates and CBRs were higher in patients treated with temsirolimus 175/75 mg compared with INV. Trends toward improvements in PFS and OS were also observed in all risk groups of patients treated with temsirolimus 175/75 mg versus INV. In all three treatment groups, patients with high risk had dismal outcomes, suggesting that MIPI classification may have value as a stratification factor for future clinical trials in relapsed and/or refractory, as well as newly diagnosed, MCL populations. Overall, this analysis indicates that earlier use of temsirolimus, when patients have favorable (low) secondary MIPI, may help to optimize its benefit.

## Design and methods

### Patients and study design

This post hoc, retrospective subset analysis utilized data from a global phase III clinical trial (ClinicalTrials.gov, NCT00117598) in which patients with relapsed or refractory MCL were randomized to receive temsirolimus 175/75 mg, temsirolimus 175/25 mg or INV. Patient recruitment and trial design were previously described [9]. This trial enrolled patients from June 2005 through July 2007. Local institutional review boards at all participating centers approved the study protocol. Participating patients provided written informed consent at the time of enrollment to permit various data analyses, including exploratory subset analyses.

### Prognostic classification

Simplified MIPI is based on four independent prognostic factors: age, Eastern Cooperative Oncology Group (ECOG) performance status, lactate dehydrogenase level and white blood cell count [13,14,17]. Performance status was collected in the database as KPS; for MIPI scoring, KPS was converted to ECOG performance status as: KPS ≥80 = ECOG 1 (0 MIPI points); KPS <80 = ECOG 2 (2 MIPI points). Characteristics of patients recorded at the time of enrollment were used to generate simplified MIPI scores and to classify patients as low, intermediate or high risk at baseline. Data were missing in some cases, resulting in a lower number of patients available for comparison. In an extensive analysis, we either excluded patients with blastoid variant, patients with missing MIPI values or patients with both blastoid variant and/or at least one missing MIPI value, or included all patients. When all patients were included, the MIPI point value for a particular missing parameter was handled as a zero. Due to the retrospective nature of this analysis, we limited the analysis to the independent assessment.

### Statistical methods

Median PFS and OS were calculated using Kaplan-Meier estimates. Significance of the treatment effect between two treatment groups is indicated by log-rank *P* values ≤0.05. The HR was calculated using a Cox proportional hazards model. Statistical analyses shown are for explanatory purposes, as the phase III trial was not powered to detect differences in outcomes by MIPI.

## Additional file

Additional file 1: Table S1. Number of patients with at least one dose delay, by simplified MIPI risk category. Number and percent of patients who had at least one dose delay by simplified MIPI risk category and by treatment arm.

## Abbreviations

CBR: Clinical benefit rate
CI: Confidence interval
CR: Complete response
ECOG: Eastern Cooperative Oncology Group
HR: Hazard ratio
INV: Investigator’s choice of therapy
KPS: Karnofsky performance status
MCL: Mantle cell lymphoma
MIPI: Mantle Cell Lymphoma International Prognostic Index
OS: Overall survival
PFS: Progression-free survival
PR: Partial response
SD: Stable disease
Std dev: Standard deviation
