# Supplementary material for: Effect of prognostic classification on temsirolimus efficacy and safety in patients with relapsed or refractory mantle cell lymphoma: a retrospective analysis
Source: Exp Hematol Oncol. 2015 Apr 11;4:11. doi: 10.1186/s40164-015-0006-1 (PMC4416347; doi:10.1186/s40164-015-0006-1)
Supplement: Additional file 1: Table S1. — Number of patients with at least one dose delay, by simplified MIPI risk category. Number and percent of patients who had at least one dose delay by simplified MIPI risk category and by treatment arm. [file 40164_2015_6_MOESM1_ESM.docx]

**Additional file 1 (online only)**

**Table S1. Number of patients with at least one dose delay, by simplified MIPI risk category** [[13](#_ENREF_13),[14](#_ENREF_14)]

| **Treatment** | **Low Risk** | **Intermediate Risk** | **High Risk** |
| --- | --- | --- | --- |
|  | **n/N (%)** | | |
| Temsirolimus 175/75 mg | 12/14 (85) | 21/24 (88) | 14/16 (88) |
| Temsirolimus 175/25 mg | 14/15 (93) | 15/18 (83) | 12/21 (57) |
| INV therapy | 10/21 (48) | 11/21 (52) | 4/11 (36) |

MIPI = Mantle Cell Lymphoma International Prognostic Index; INV = Investigator’s choice; n = number of patients with at least one dose delay; N = total number of patients in the risk category.
